# Anticancer Effect of Polyphyllin I in Suppressing Stem Cell-Like Properties of Hepatocellular Carcinoma via the AKT/GSK-3*β*/*β*-Catenin Signaling Pathway

**DOI:** 10.1155/2022/4031008

**Published:** 2022-10-22

**Authors:** Mianmian Liao, Haiyan Du, Bing Wang, Jinzhen Huang, Danping Huang, Guangdong Tong

**Affiliations:** ^1^Department of Hepatology, Shenzhen Traditional Chinese Medicine Hospital, The Fourth Clinical Medical College of Guangzhou University of Chinese Medicine, Shenzhen, China; ^2^Nanjing University of Traditional Chinese Medicine, Nanjing, China; ^3^Department of Integrated Traditional Chinese and Western Medicine, School of Clinical Medicine of Guangdong Pharmaceuticcal University, Guangzhou, China

## Abstract

Polyphyllin I (PPI), also called Chong Lou saponin I, is a steroidal saponin isolated from the rhizome of *Paris polyphylla*. PPI has been demonstrated to have strong anticancer activity. However, its effect on the stemness of liver cancer stem cells (LCSCs) is not completely understood. Herein, we aimed to investigate the effect of PPI on the stem cell-like features of LCSCs and hepatocellular carcinoma (HCC). LCSCs were enriched in a serum-free medium and treated with PPI, sorafenib (Sora), or PPI and Sora. Several endpoints, including spheroid formation and differentiation, cell proliferation, surface markers of LCSCs, PPI binding targets, and stemness-associated protein expression, were evaluated. Immunofluorescence staining, quantitative real-time polymerase chain reaction, siRNA transfection, and coimmunoprecipitation ubiquitination assays were conducted for in-depth mechanistic studies. Evaluation of *in vivo* antitumor efficacy demonstrated that PPI effectively inhibited the proliferation of liver cancer cells and the self-renewal and differentiation of LCSCs. Flow cytometry indicated that PPI suppressed the expression of the stem cell surface markers EpCAM and CD13. Molecular docking showed a high affinity between PPI and proteins of the Wnt/*β*-catenin signaling pathway, including AKT, GSK-3*β*, and *β*-catenin, with the binding energies of -5.51, -5.32, and -5.40 kcal/mol, respectively, which suggested that PPI might regulate the Wnt/*β*-catenin signaling pathway to affect the stem cell-like properties of HCC. Further *ex vivo* experiments implied that PPI activated the AKT/GSK-3*β*-mediated ubiquitin proteasomal degradation of *β*-catenin and subsequently attenuated the prooncogenic effect of LCSCs. Finally, the anticancer property of PPI was confirmed *in vivo*. It was found that PPI inhibited the tumor growth in an HCC cell line xenograft model. Taken together, molecular docking analysis and experimental data highlighted the novel function of PPI in suppressing the stem cell-like characteristics of LCSCs via the AKT/GSK-3*β*/*β*-catenin signaling pathway.

## 1. Introduction

Cancer is a leading cause of death worldwide, accounting for 10 million deaths in 2020 [1], and primary liver cancer is the third leading cause of cancer-related mortality, with 830,000 deaths worldwide [2]. Hepatocellular carcinoma (HCC) accounts for 75-85% of the total cases of primary liver cancer [3]. Most patients with HCC are diagnosed at an advanced stage, which leads to a poor prognosis and a loss of the opportunity for surgery. Although great progress has been made in treatment strategies, such as surgical resection, transarterial chemoembolization, radiofrequency ablation, and liver transplantation, serious toxic side effects, drug resistance, and drug inefficacy in advanced-stage HCC remain the main obstacles for these therapies [4, 5]. Hence, it is vital to explore new strategies for HCC treatment.

Cancer stem cells (CSCs) in HCC are characterized by continuous self-renewal, differentiation, and tumorigenic capabilities, which are considered the cause of therapeutic resistance, tumor emergence, recurrence, and metastasis [6, 7]. Although LCSCs only represent a small subset of liver cancer cells, they are considered to be responsible for HCC initiation, propagation, maintenance, metastasis, recurrence, and resistance to chemotherapy and radiotherapy [8, 9]. Targeting LCSCs is therefore a promising therapeutic strategy to improve the survival or quality of life of patients with HCC.

However, the mechanistic aspects of LCSC-mediated HCC development are relatively unknown. Studies have revealed that Wnt signaling proteins play an important role in the culture of CSCs [10, 11]. LCSCs have been characterized based on the expression of various stem cell markers, including CD133, CD13, CD90, EpCAM, NANOG, and OCT-4 [12, 13]. Currently, the specific markers for LCSCs are still debated in the field. Cao et al. confirmed CD13 as a marker for CSCs in HCC, which plays an important role in LCSC regulation [14]. EpCAM, which regulates the self-renewal of CSCs in HCC, has also been defined as an LCSC marker [15, 16].

Traditional Chinese medicine has been practiced for thousands of years and has become a promising strategy for cancer therapy, with minimal side effects and a high efficiency. *Paris polyphylla* (Chong Lou) is a well-known medicinal plant that has been used in various traditional Chinese formulations for wound healing and anticancer therapy [17, 18]. Polyphyllin I (PPI) is a steroidal saponin extracted from Chong Lou [19]. Previous reports have revealed that PPI exerts anticancer effects against a variety of cancers by inhibiting the proliferation, invasion, apoptosis, drug resistance, and immune function of tumor cells [20–26]; therefore, PPI has been used as a possible candidate of anticancer drug. However, the mechanisms by which PPI restricts the self-renewal of LCSCs remain largely unknown.

In this study, we found that PPI inhibited the proliferation of liver cancer cells and limited the self-renewal and differentiation ability of LCSCs, possibly via proteasomal degradation of *β*-catenin. PPI was also found to restrain xenograft tumor growth in nude mice. Taken together, the results of our study provide supporting evidence for the anti-LCSC activity of PPI against HCC.

## 2. Materials and Methods

### 2.1. Chemicals

**Figure 1:**
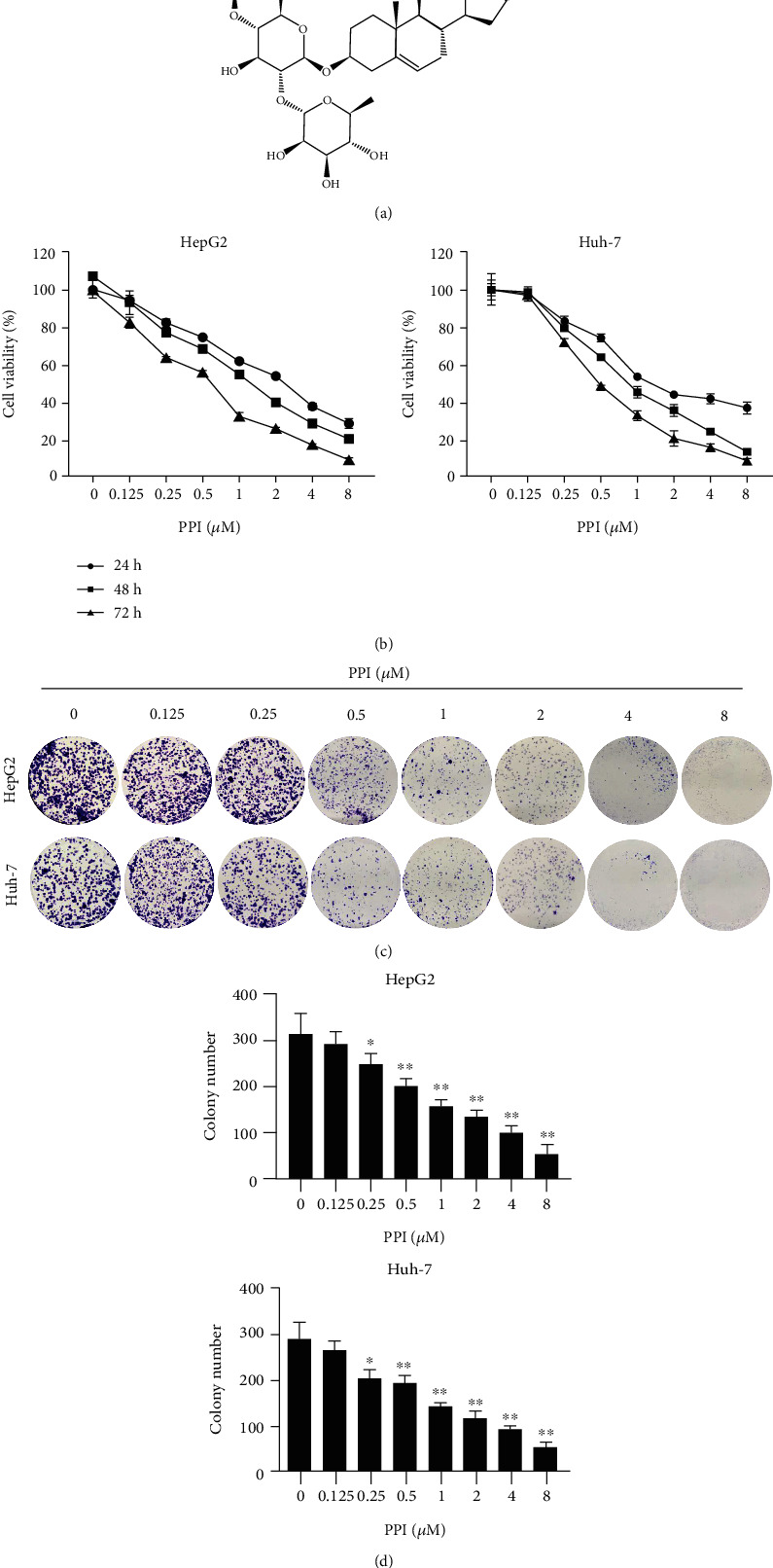
PPI suppressed the proliferation of HCC cells. (a) Chemical structure of polyphyllin I. (b) A CCK-8 assay was performed to assess the inhibition ability of PPI on the proliferation of HepG2 and Huh-7 cells after incubation for 24, 48, and 72 h. (c) A colony formation assay was used to observe the long-term inhibitory effects of PPI against HCC cell lines. (d) Results of statistical analysis of the clonogenic assay. ^∗^*P* < 0.05 and ^∗∗^*P* < 0.01*vs.* the control group.

PPI (S9114), sorafenib (Sora; S7397), and SC79 were purchased from Selleck Chemicals (Shanghai, China). Cycloheximide (CHX; 508739) and MG132 (474790) were obtained from Sigma–Aldrich (St. Louis, MO, USA). Dimethyl sulfoxide was used to dissolve PPI and Sora, which were then stored at -20°C. The structure of PPI is displayed in Figure 1(a).

### 2.2. Cell Culture

The HepG2 human hepatoma cell line was obtained from ATCC and maintained according to the ATCC guidelines. The human HCC cell line Huh-7 was purchased from MEIXUAN Biological Science and Technology (Shanghai, China). HepG2 cells were cultured in Eagles Minimum Essential Medium (EMEM; ATCC) with 10% fetal bovine serum (FBS; Gibco) and 1% penicillin and streptomycin (P/S; Gibco). RPMI-1640 medium (Gibco) with 10% FBS and 1% P/S was used to culture Huh-7 cells. LCSCs were cultured in DMEM/F12 medium (Gibco), with B27 (Invitrogen), insulin (Sigma), recombinant human epidermal growth factor (BD Biosciences), 1% P/S, and 0.4% bovine serum albumin (BSA; Solarbio). All cells were cultured at 37°C in a humidified incubator with 5% CO_2_.

### 2.3. Cell Viability Assay

The cytotoxicity of PPI was detected using Cell Counting Kit-8, (CCK-8; KeyGen, China). HepG2 and Huh-7 cells were seeded in 96-well plates and incubated overnight. The cells were then treated with PPI (0, 0.125, 0.25, 0.5, 1, 2, 4, and 8 *μ*M) for 24, 48, and 72 h. Finally, the CCK-8 reagent was added to each well, and the plates were incubated in a humidified incubator for 3 h. Half-maximal inhibitory concentration (IC_50_) values were calculated, and cell proliferation curves for both HepG2 and Huh-7 cells were plotted.

### 2.4. Colony Formation Assay

For a colony formation assay, HepG2 and Huh-7 cells were seeded in 6-well plates and incubated overnight for cell attachment, followed by the intervention with PPI at different concentrations (0, 0.125, 0.25, 0.5, 1, 2, 4, and 8 *μ*M). The cells were then cultured for 2 weeks in fresh complete media to gain a further insight into the long-term effects of PPI. Subsequently, cell colonies were fixed with 4% paraformaldehyde and stained with 0.5% crystal violet (Beyotime Biotechnology).

### 2.5. Apoptosis Assay

HepG2 and Huh-7 cells were seeded in 24-well plates with 500 *μ*L of complete culture media, cultured overnight, and then treated with PPI or Sora for 48 h. Hoechst 33258 staining was used to observe the morphological features of LCSCs during apoptosis. Briefly, 100 *μ*L of Hoechst 33258 staining solution was added to the plates for 30 min, then gently aspirated, and the plates were washed with 1x phosphate-buffered saline (PBS) three times. Images were directly observed using a fluorescence microscope.

### 2.6. Sphere Formation and Differentiation Assay

LCSCs derived from HepG2 and Huh-7 cells were selectively cultivated in the CSC culture medium in 6-well ultralow-adhesion plates. The medium was exchanged with a fresh complete cell medium using the half-change method every other day. PPI, Sora, and PPI plus Sora were added to the CSC culture medium to investigate their effects on the formation of tumor spheres. The size and number of primary spheres were assessed by microscopy. Lastly, following 10 days of culture, CSC spheres were cultured in 6-well plates with complete medium for 48 h to determine their differentiation ability and observe morphological changes by microscopy.

### 2.7. Flow Cytometry Analysis

HepG2 and Huh-7 cells were seeded at a density of 3 × 10^5^ cells/well in 6-well plates and cultured overnight. After treatment with PPI, Sora, or PPI plus Sora for 48 h, the cells were digested, rinsed, and resuspended. EpCAM-FITC and CD13-PE antibodies (BD Biosciences) were added to the cell suspensions, and the mixtures were incubated for 30 min in the dark. FITC- and PE-labeled isotype IgG1 antibodies served as negative controls. Subsequently, the cells were washed with 1x PBS three times and measured using a BD LSRFortessa flow cytometer (BD Biosciences). The results were analyzed using the FlowJo software.

### 2.8. Molecular Docking

PPI interactions with and binding affinities for *β*-catenin, p53, PI3K, PPAR*α*, AKT, and GSK-3*β* were analyzed using the AutoDock v4.2.6 and AutoDock-Tools v1.5.6 software. The three-dimensional structure of PPI was downloaded from PubChem (https://pubchem.ncbi.nlm.nih.gov/), and the crystal structures of *β*-catenin, p53, PI3K, PPAR*α*, AKT, and GSK-3*β* were obtained from the RCSB Protein Data Bank (http://www.rcsb.org/pdb/). Before docking, the PPI and protein structures were prepared by adding polar hydrogen atoms, computing the Gasteiger charge, and assigning the AD4 atom type. A grid box with a size of 60 × 60 × 60 Å and a grid spacing at 0.375 Å that was centered on the ligand were used to cover the residues at the binding site. Finally, docking between PPI and *β*-catenin, p53, PI3K, PPAR*α*, AKT, and GSK-3*β* were performed using Lamarck's genetic algorithm. Parameters including the number of individuals in the population, maximum number of energy evaluations, and maximum number of generations were studied, with the number of GA runs set to 150, 5.0 × 10^6^, 2.7 × 10^4^, and 50. The Discovery Studio client and PyMOL software were used to analyze and visualize the docking results.

### 2.9. Quantitative Real-Time Polymerase Chain Reaction (qPCR)

Isolation and reverse transcription of total RNA were performed according to standard procedures. qPCR was carried out using a Bio-Rad ViiA7 system, a reverse transcription-PCR kit, and a SYBR Green kit (Takara). Calculations of relative RNA levels were performed using the comparative Ct method (*ΔΔ*Ct). The selected *β*-actin and *β*-catenin primers were designed by Sangon Biotech. The primer sequences were as follows: *β*-actin (F) 5′-TCCTTCCTGGGCATGGA-3′ and (R) 5′-AGGAGGAGC-AATGATCTTGATCTT-3′ and *β*-catenin (F) 5′-ACAGCACCTTCAGCACTCT-3′ and (R) 5′-AAGTTCTTGGCTATTACGACA-3′.

### 2.10. Western Blotting Analysis

LCSC spheres were treated with PPI, Sora, and PPI plus Sora for 48 h, followed by lysis with radioimmunoprecipitation assay buffer (Beyotime) supplemented with a protease inhibitor mixture (Roche Diagnostics) for 30 min at 4°C. Total protein concentrations were measured using a protein quantitation kit (KeyGen Biotech). Subsequently, equal amounts of protein were subjected to 10% SDS-PAGE, and the proteins were transferred to polyvinylidene fluoride microporous membranes (Millipore). The membranes were then blocked with 5% skim milk for 2 h and incubated with primary antibodies at 4°C overnight, followed by incubation with the respective secondary antibodies (Abcam) for 1 h at room temperature (RT). The following primary antibodies were used: NANOG, OCT-4, AKT, p-AKT, GSK-3*β*, p-GSK-3*β*, total *β*-catenin, nuclear *β*-catenin, cytoplasmic *β*-catenin, LAMB1, and *β*-actin (CST). The protein bands were then transferred to enhanced chemiluminescence reagents (Millipore) and imaged using a Tanon detection system. Band intensities were quantified using the ImageJ software.

### 2.11. Coimmunoprecipitation (Co-IP) Ubiquitylation Analysis

LCSCs were treated with PPI for 48 h, and Co-IP ubiquitination analysis was performed using the Capturem™ IP and Co-IP kit (Takara) according to the manufacturer's instructions. Samples of precipitated input controls and proteins were subjected to western blot analysis with the corresponding antibodies, as indicated in the previous section.

### 2.12. Immunofluorescence Staining

Cells were seeded in climbing slices and treated with PPI or Sora for 48 h, followed by fixation for 1 h. The cells were then blocked and permeabilized using 5% BSA (Sigma) in 0.1% Triton X-100/PBS for 2 h at RT, followed by incubation with a primary antibody (*β*-catenin, 1 : 100, CST) for 2 h at RT and washed with 1x PBS three times for 5 min. The cells were then incubated with a goat anti-rabbit IgG H&L Alexa Fluor 488 secondary antibody (1 : 100, CST) for 1 h in the dark. The cell climbing slices were washed three times for 5 min each with 1x PBS. Staining was performed 2-(4-amidinophenyl)-6-indolecarbamidine dihydrochloride (DAPI) for 15 min at RT under darkness. The slides were sealed with antifluorescence quenching tablets (Solarbio) after cell climbing. For image acquisition, a Zeiss LSM laser scanning confocal microscope was used with 405, 488, and 570 nm excitation, and images were analyzed using the Zen software (Zeiss).

### 2.13. Transient Transfection

*β*-Catenin and nonspecific siRNAs were constructed by GenePharma. Transfection of the siRNAs was performed with the HiPerFect transfection reagent (HanBio Biotechnology). First, 3 × 10^5^ cells were seeded in a 6-well plate with 2 mL of complete medium. Next, the siRNAs or NC were mixed with the transfection reagent, and the mixtures were incubated at RT for 10 min. Subsequently, the complexes were transfected into LCSCs for 24 h, and the transfection reagent was replaced by a fresh complete medium.

### 2.14. Animal Experiment

Twenty-four male BALB/c nude mice were purchased from the Medical Experiment Center in Guangdong Province to study the inhibitory effect of PPI on tumor growth *in vivo*. This research was approved by the Animal Care and Use Committee at Guangzhou University of Chinese Medicine. Approximately 5 × 10^6^ Huh-7 CSCs with Dual Luciferase were resuspended in 100 *μ*L of Matrigel (Corning) and injected into the right flanks of the mice. The mice were randomly assigned to each experimental group, including control (equal volume of saline), Sora (30 mg/kg), PPI (1 mg/kg), and PPI (1 mg/kg) combined with Sora (30 mg/kg) groups. Body weights and tumor volumes were measured and recorded every 3 days. D-luciferin (PerkinElmer) was injected intraperitoneally for the acquisition of tumor optical images using the IVIS Lumina XR imaging system (PerkinElmer). The mice were sacrificed when the tumors in the control group reached 20 × 20 mm. Tumors and other organs were then collected from the four groups for hematoxylin and eosin (H&E) staining and immunohistochemistry (IHC).

### 2.15. H&E Staining and IHC Analysis

H&E staining and IHC analysis were performed to further assess the anticancer efficacy of PPI *in vivo*. Xenograft tumors were dehydrated and embedded in paraffin, and serial sections with a slice thickness of 4 *μ*m were prepared. Samples were then dewaxed and hydrated according to a standard procedure. For H&E staining, 10% hematoxylin was used to stain the cell nuclei for 5 min, and eosin was used to counterstain the cytoplasm for 2 min. The stained sections were then dehydrated, hyalinized, and sealed with neutral gum. For IHC, methanol with 0.3% hydrogen peroxide was used to block endogenous peroxidase activity, and goat serum was used to decrease nonspecific binding. The sections were then incubated with primary antibodies against KI67, NANOG, OCT-4, and *β*-catenin at 4°C for 12 h, and a DAB substrate–chromogen solution was applied for color development after incubation with secondary antibodies. The sections were counterstained with hematoxylin and sealed with neutral gum. Finally, the sections were photographed and stored.

### 2.16. Statistical Analysis

Data are presented as the mean ± standard deviation. All statistical analyses were conducted using the SPSS software (version 19.0) and plotted with GraphPad Prism 7.0. An independent-samples *t*-test was used for two groups of data in accordance with independence, normality, and homogeneity of variance, and one-way analysis of variance was performed for comparison among multiple groups. *P* < 0.05 was considered statistically significant.

## 3. Results and Discussion

### 3.1. PPI Suppressed the Proliferation of HCC Cells

The CCK-8 assay was conducted to assess the inhibitory effect of PPI on HepG2 and Huh-7 cells. The HCC cell lines were treated with PPI for 24, 48, and 72 h. IC_50_ values of 2.177 and 2.094 *μ*M were obtained for HepG2 and Huh-7 cells, respectively, after treatment with PPI for 24 h, then decreased to 1.298 and 1.010 *μ*M, respectively, when exposure was prolonged to 48 h, and to 0.5543 and 0.58 *μ*M, respectively, after 72 h (Figure 1(b)). These results indicated that PPI suppressed the proliferation of HepG2 and Huh-7 cells in both dose- and time-dependent manners. A colony formation assay was then performed to assess the long-term inhibitory effects of PPI treatment on the HCC cell lines. The results showed that the colony numbers and sizes decreased for HepG2 and Huh-7 cells as the PPI concentration increased (Figures 1(c) and 1(d)). Overall, PPI treatment was found to effectively inhibit the proliferation of both HepG2 and Huh-7 cells.

### 3.2. PPI Limited the Self-Renewal Ability of LCSCs

**Figure 2:**
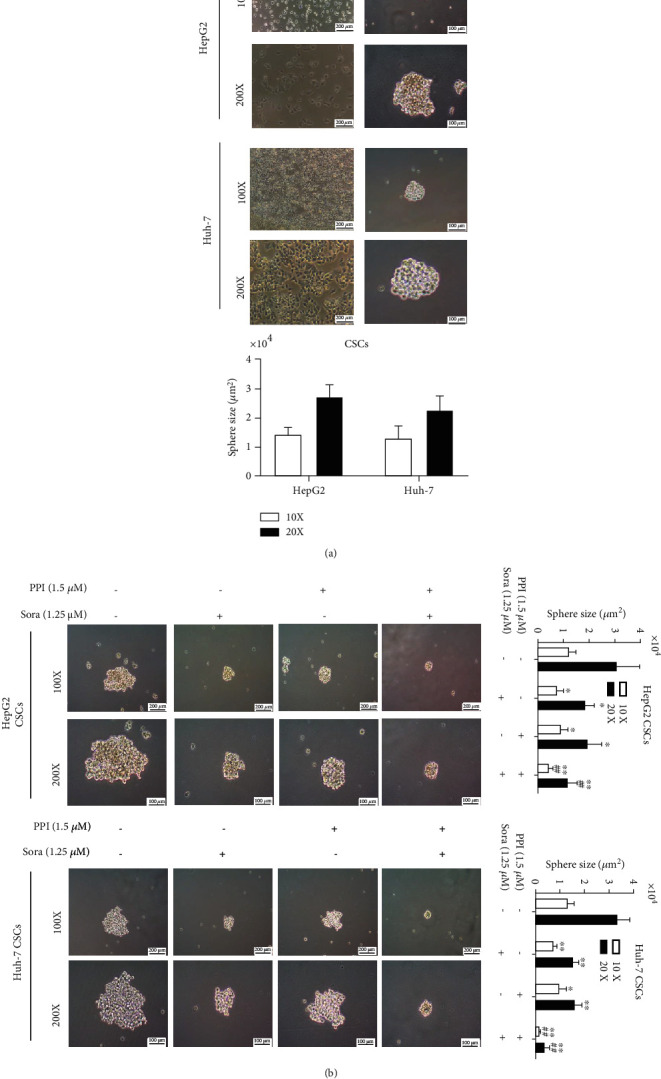
PPI limited the self-renewal ability of LCSCs. (a) Enrichment of LCSCs derived from HepG2 and Huh-7 cells using a serum-free suspension culture and morphological comparison of HCC cells and LCSCs. (b) A tumor sphere formation assay was carried out to test the inhibitory effect of PPI on the self-renewal ability of LCSCs. ^∗^*P* < 0.05 and ^∗∗^*P* < 0.01*vs.* the control group; ^#^*P* < 0.05 and ^##^*P* < 0.01*vs.* the Sora group.

The spheres of LCSCs derived from HepG2 and Huh-7 cells were enriched by incubation in a serum-free medium. A microsphere formation assay was used to investigate the inhibitory effect of PPI on the self-renewal ability of the spheres. As shown in Figure 2(a), LCSC spheres were successfully enriched. After PPI addition, with or without Sora, to the SFM, the self-renewal ability of LCSCs was suppressed, and the combination of PPI and Sora more effectively decreased the numbers and sizes of LCSC spheres (Figure 2(b)).

### 3.3. PPI Suppressed the Differentiation Capability and Promoted Apoptosis of LCSCs

**Figure 3:**
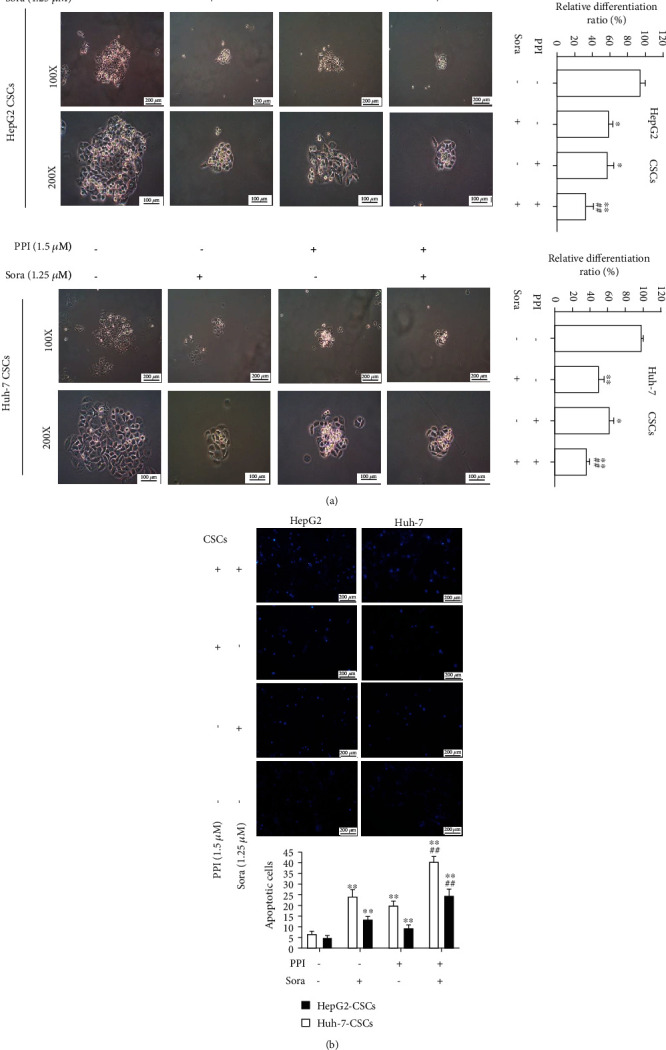
PPI suppressed the differentiation capability and promoted the apoptosis of LCSCs. (a) A tumor sphere differentiation experiment was performed to evaluate the effect of PPI on the differentiation potential of LCSCs. (b) Hoechst 33258 staining was used to detect the inhibition of LCSC proliferation by PPI. ^∗^*P* < 0.05 and ^∗∗^*P* < 0.01*vs.* the control group; ^#^*P* < 0.05 and ^##^*P* < 0.01*vs.* the Sora group.

Considering that the multidifferentiation potential is an important feature of CSCs, we further investigated whether PPI could limit the differentiation ability of LCSCs. Microspheres that are grown in serum-free medium are capable of redifferentiating into adherent cells when cultured in a complete medium that contains serum, which is also referred to CSC differentiation. We therefore reseeded 10-day-old LCSC spheres into 6-well plates with a complete medium containing PPI, Sora, or a combination of PPI and Sora. PPI and Sora were both found to suppress differentiated LCSC clusters, while the combination group exerted the best inhibitory effect (Figure 3(a)). Hoechst 33258 dye staining was conducted to detect the inhibition of LCSC proliferation by the treatment with PPI or Sora. The nuclei of apoptotic cells were stained with diluted Hoechst 33258 for 20 min, and analysis demonstrated that both PPI and Sora accelerated the number of apoptotic LCSCs after administration for 48 h, as shown in Figure 3(b). The inhibitory effect on the proliferation was more obvious in the group that received PPI with Sora.

### 3.4. PPI Reduced the Proportion of LCSCs

**Figure 4:**
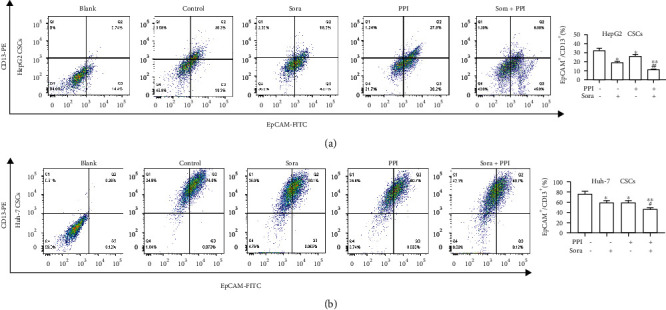
PPI reduced the proportion of LCSCs. (a, b) PPI reduced the proportion of the LCSC surface markers EpCAM and CD13. ^∗^*P* < 0.05 and ^∗∗^*P* < 0.01*vs.* the control group; ^#^*P* < 0.05 and ^##^*P* < 0.01*vs.* the Sora group.

Numerous studies have proposed that CD13 and EpCAM, as cellular surface and clinical prognostic markers, could be used to identify LCSCs [27, 28]. EpCAM is a common CSC marker in various cancers, while the EpCAM^+^CD13^+^ phenotype is known to indicate the presence of LCSCs. EpCAM together with CD13 was thus selected as the cell surface marker for LCSCs in this study. Changes in the specific populations of LCSCs were determined by flow cytometry before and after treatment with PPI and Sora. As shown in Figure 4(a), both PPI and Sora decreased the proportion of EpCAM^+^CD13^+^ cells in HepG2 CSCs, with the subpopulations of EpCAM^+^CD13^+^ cells in the PPI, Sora, and PPI plus Sora groups reduced to 18.0%, 27.9%, and 9.88%, respectively, compared with that observed in the control group (30.3%). Similar effects were observed for Huh-7 CSCs, with the groups that received PPI, with or without Sora, displaying relatively lower EpCAM^+^CD13^+^ expression than the control group (Figure 4(b)). The proportions of EpCAM^+^CD13^+^ cell subpopulations in the PPI, Sora, and PPI plus Sora groups were 60.1%, 60.7%, and 48.7%, respectively, while those in the Huh-7 CSCs control group reached 74.0%. These results indicated that PPI could potently suppress the proliferation of LCSCs, which might contribute to tumorigenesis and tumor recurrence of HCC.

### 3.5. Prediction of the PPI Targets Based on Molecular Docking

**Figure 5:**
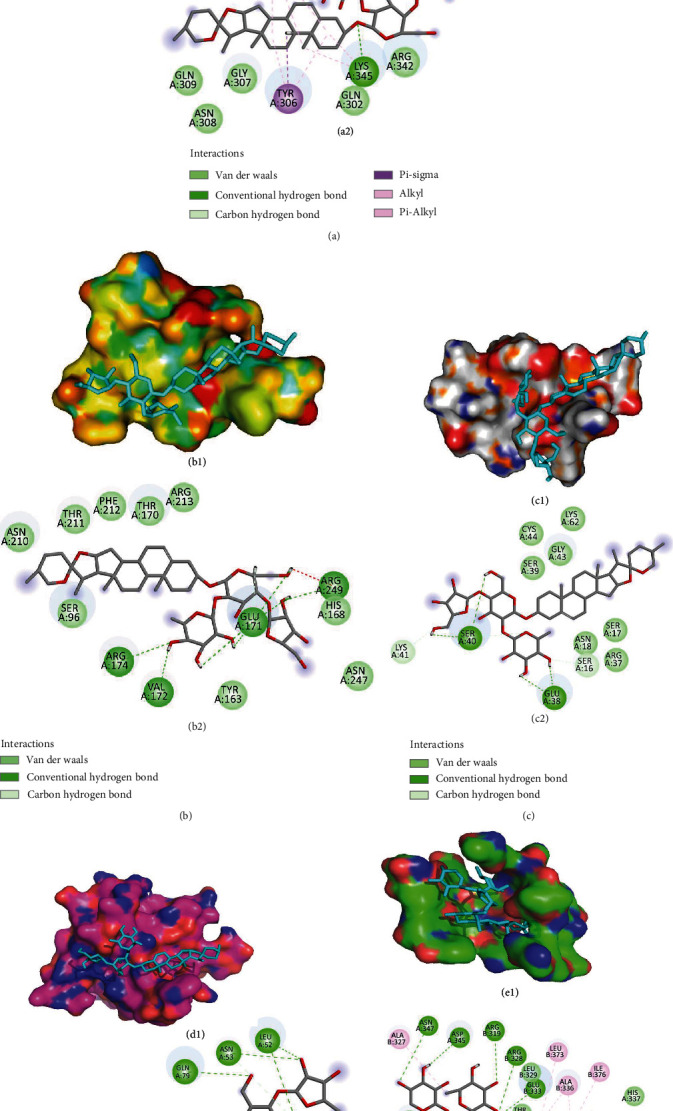
Prediction of the PPI target based on molecular docking (A1, A2) Interaction between PPI and *β*-catenin. (B1, B2) Interaction between PPI and p53. (C1, C2) Interaction between PPI and PI3K. (D1, D2) Interaction between PPI and AKT. (E1, E2) Interaction between PPI and GSK-3*β*. Residues around the compound are displayed at the surface (A1–E1), and the interaction mode is shown in 2D diagrams (A2–E2). PPI is colored in cyan.

As reported previously [29], the signaling pathways of *β*-catenin, p53, PI3K, and PPAR*α* might be the specific targets of PPI and be associated with the antitumor propensity of PPI in various cancers. Moreover, researches have suggested that the *β*-catenin, p53, PI3K, and PPAR*α* signaling pathways are responsible for regulating cancer cell stemness [30–32]. Molecular docking was therefore performed to determine whether PPI exerts antitumor activity by interfering with these pathways. The molecular docking information is provided in Table 1, and the interaction diagrams are provided in Figures 5(a)–5(c). As shown in Table 1, PPI was calculated to bind to *β*-catenin, p53, and PI3K with binding energies of -5.4, -5.07, and -4.66 kcal/mol, respectively. The results showed that PPI could more preferably bind to *β*-catenin, with the lowest docking score (-5.4 kcal/mol) and the highest interaction. However, the binding energy of +3.98 kcal/mol between PPI and PPAR*α* indicated that PPI did not bind well to PPAR*α*. To further identify whether PPI could regulate the Wnt/*β*-catenin signaling pathway in LCSCs, dockings between PPI and AKT and GSK-3*β* were conducted. We found that PPI was able to bind to AKT and GSK-3*β* with binding energies of -5.51 and -5.32 kcal/mol (Table 1, Figures 5(d) and 5(e)). Taken together, the molecular docking results suggested that PPI might regulate the Wnt/*β*-catenin signaling pathway to affect the stem cell-like properties of cancer cells.

### 3.6. PPI Affected the AKT/GSK-3*β*/*β*-Catenin Signaling Pathway in LCSCs

**Figure 6:**
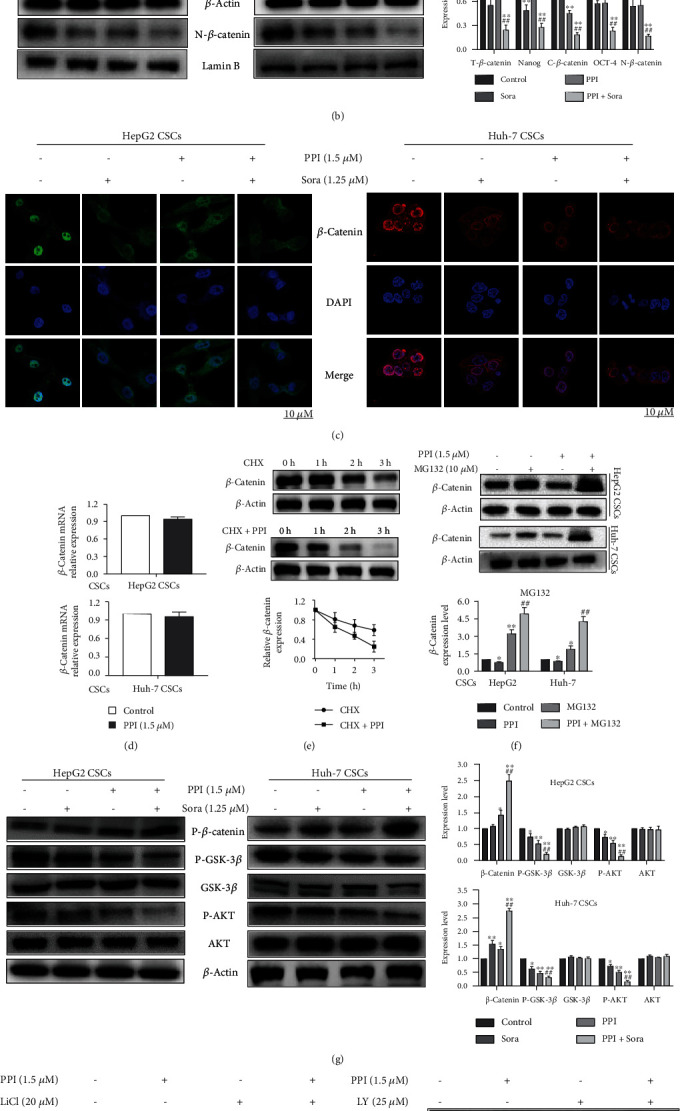
PPI affected the AKT/GSK-3*β*/*β*-catenin signaling pathway in LCSCs. (a) Western blotting was performed to compare the protein expression of *β*-catenin, NANOG, and OCT-4 in HCC cells and LCSCs. (b) PPI suppressed the protein expression of *β*-catenin, NANOG, and OCT-4 in LCSCs. (c) Immunofluorescence analysis was conducted to detect nuclear accumulation of *β*-catenin in PPI- and Sora-treated LCSCs. (d) A qPCR assay was conducted to examine the mRNA level of *β*-catenin in LCSCs after exposure to PPI. (e) *β*-catenin downregulation was triggered in the CHX group, while its degradation was remarkably facilitated in the CHX+PPI group. (f) The expression of *β*-catenin could not be downregulated by PPI whenMG132 was added. ^∗^*P* < 0.05 and ^∗∗^*P* < 0.01*vs.* the control group; ^#^*P* < 0.05 and ^##^*P* < 0.01*vs.* the MG132 group. (g) Western blotting demonstrated that PPI administration notably affected the main proteins of the AKT/GSK-3/*β*-catenin signaling pathway. (h) Western blotting indicated that PPI reversed the promoting effect of LiCl on the *β*-catenin level. The expression of *β*-catenin in the LCSCs was further decreased by PPI in the presence of LY (the AKT inhibitor) treatment. Meanwhile, inhibition of *β*-catenin by PPI is rescued by SC79 (the Akt activator). ^∗^*P* < 0.05 and ^∗∗^*P* < 0.01*vs.* the control group; ^#^*P* < 0.05 and ^##^*P* < 0.01*vs.* the Sora group.

Accumulating evidence has demonstrated that *β*-catenin plays a crucial role in maintaining CSC activity [33]. Thus, we further verified whether the anticancer function of PPI depends on *β*-catenin. First, we examined the differential expression of *β*-catenin, the typical CSC markers NANOG and OCT-4 in HepG2 cells, Huh-7 cells, and LCSCs. As shown in Figure 6(a), the expression levels of *β*-catenin, NANOG and OCT-4 were higher in LCSCs than in HepG2 and Huh-7 cells, indicating that *β*-catenin was involved in the regulation of LCSC growth. *β*-Catenin is the central component of the Wnt/*β*-catenin pathway. The activation of the Wnt/*β*-catenin pathway is mainly dependent on cellular redistribution and nuclear accumulation of *β*-catenin [34, 35]. Therefore, we determined the effect of PPI on the expression and subcellular localization of *β*-catenin. As indicated in Figure 6(b), both total and fractional expression levels of *β*-catenin were downregulated when LCSCs were treated with PPI or Sora. The immunofluorescence assay corroborated these findings, showing a reduction in the nuclear accumulation of *β*-catenin when LCSCs were treated with PPI or Sora (Figure 6(c)). Next, the mRNA level of *β*-catenin in LCSCs after PPI treatment was determined by qPCR. The results (Figure 6(d)) suggested that the mRNA level of *β*-catenin did not change after LCSC exposure to PPI. To further elucidate the mechanism by which PPI regulates the level of *β*-catenin, either the proteasome inhibitor MG132 or the protein synthesis inhibitor CHX was added to the LCSC medium. As shown in Figure 6(e), LCSCs treated with PPI exhibited an increased *β*-catenin degradation rate compared with those in the control group in the presence of CHX, indicating that PPI facilitated *β*-catenin protein degradation. It was reported that proteasome-mediated degradation of *β*-catenin was the main mechanism responsible for *β*-catenin degradation [36]. A proteasome inhibitor, MG132, was added to block proteasome-mediated protein degradation. The results presented in Figure 6(f) indicated that PPI could not downregulated the expression of *β*-catenin in both HepG2 and Huh-7 CSCs when the proteasome pathway was blocked. Based on these results, PPI downregulated *β*-catenin depended on the activation of proteasomal degradation pathway.

Additionally, it has been suggested that the AKT/GSK-3*β* pathway is a critical functional signaling pathway involved in the regulation of *β*-catenin and CSC activity [37]. Stimulation of the canonical Wnt/*β*-catenin signaling pathway through specific ligands deactivates GSK-3*β via* specific phosphorylation at Ser9, stabilizes *β*-catenin, and favors its translocation to the nucleus.  Figure 6(g) shows that both PPI and Sora diminished the phosphorylation of GSK-3*β* (Ser9), suggesting that PPI is a negative factor in *β*-catenin stabilization. Furthermore, available data suggest that GSK-3*β* is a downstream target of AKT, and the inhibition of GSK-3*β* phosphorylation at Ser9 as a result of PPI treatment might depend on AKT. We found that p-AKT was also significantly suppressed by PPI in LCSCs (Figure 6(g)), indicating that AKT inhibition might facilitate the PPI-mediated *β*-catenin degradation. To thoroughly investigate whether AKT/GSK3*β* signaling is responsible for *β*-catenin degradation in the PPI treatment group, the AKT inhibitor LY294002 (LY), AKT activator SC79 and the GSK-3*β* inhibitor LiCl were used to suppress the activity of AKT and GSK-3*β*, respectively. The result of following experiments showed that the expression of *β*-catenin in the LCSCs was further decreased by PPI in the presence of LY treatment, and the inhibition of *β*-catenin expression by PPI is rescued by SC79 (the Akt activator). Meanwhile, PPI could offset the *β*-catenin-enhanced effect of LiCl partially (Figure 6(h)).

### 3.7. PPI Suppressed the Stem Cell-Like Properties of HCC via *β*-Catenin Proteasomal Degradation

**Figure 7:**
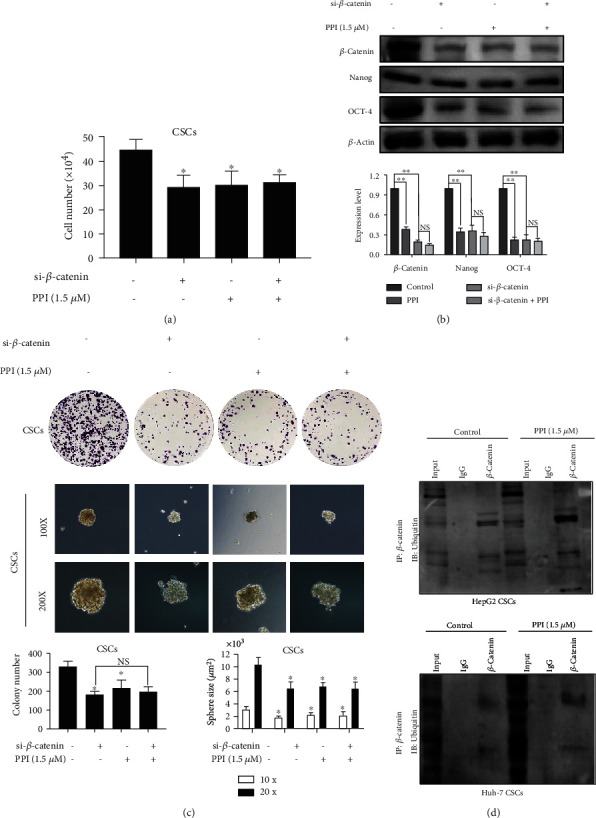
PPI suppressed the stem cell-like properties of HCC via proteasomal degradation of *β*-catenin. (a) A cell-counting assay revealed that PPI could not significantly limit the number of LCSCs with *β*-catenin silencing. (b) Western blotting indicated that PPI treatment could not significantly suppress the expression of *β*-catenin, NANOG, and OCT-4 on LCSCs with *β*-catenin silencing. (c) Colony formation analysis and a sphere formation assay showed that PPI have no significant long-term inhibitory effects of PPI on LCSCs with *β*-catenin silencing. (d) Co-IP and ubiquitination experiments showed that the ubiquitination status of *β*-catenin in LCSCs was notably upregulated by PPI administration. ^∗^*P* < 0.05 and ^∗∗^*P* < 0.01*vs.* the control group; ^#^*P* < 0.05 and ^##^*P* < 0.01*vs.* the Sora group.

To confirm whether the critical role of *β*-catenin is essential in mediating LCSC stemness by PPI, the *β*-catenin level was downregulated by transfecting its siRNA to CSCs with a lentivirus vector. The results of cell counting, western blotting, the tumor sphere assay, and the colony formation experiments demonstrated that *β*-catenin silencing could effectively inhibit not only the expression of *β*-catenin but also the proliferation and self-renewal capacity of LCSCs and the expression of the cancer stemness markers NANOG and OCT-4 (Figures 7(a)–7(c)). Meanwhile, no significant reduction was observed in the PPI combined with siRNA group compared with that in the PPI-alone or the siRNA-alone group, indicating that PPI might abolish the stem cell-like properties of HCC by modulating the stability of *β*-catenin. Since proteasome-mediated degradation of *β*-catenin is the major pathway responsible for *β*-catenin degradation [38], we next performed Co-IP combined with a ubiquitination assay to further elucidate the mechanism by which PPI regulates the level of *β*-catenin. As shown in Figure 7(d), the intensity of ubiquitinated *β*-catenin protein in the PPI-untreated group was much less than that in the PPI-treated group, showing that PPI might promote the ubiquitination process of *β*-catenin.

### 3.8. PPI Repressed the Xenograft Tumor Growth and Stem Cell-Like Properties of HCC *In Vivo*

**Figure 8:**
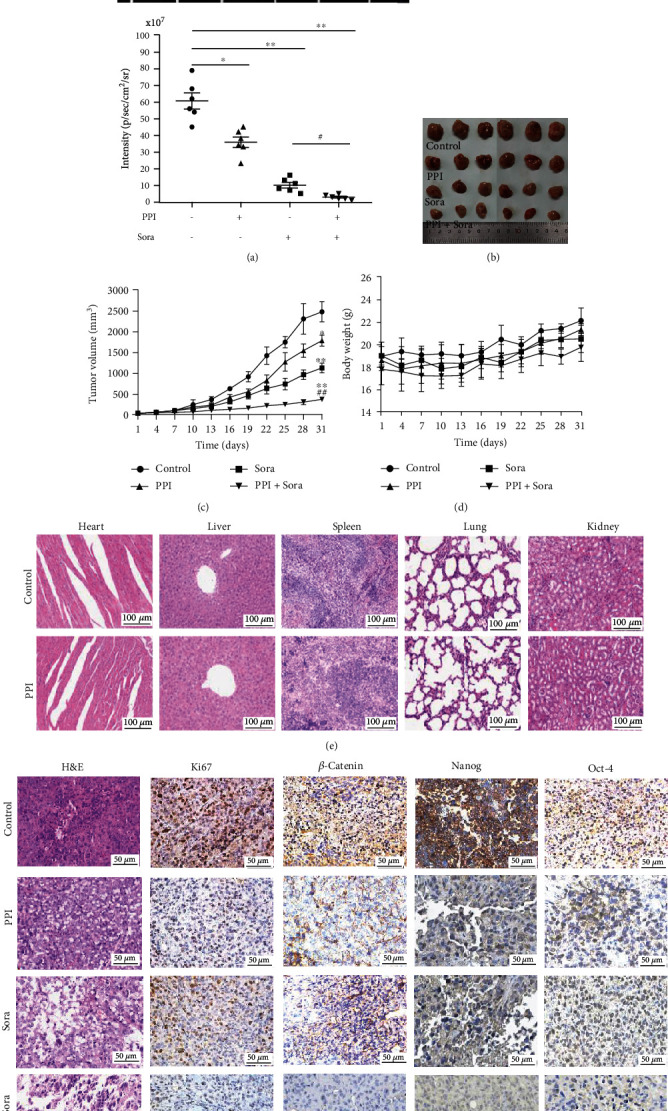
PPI repressed xenograft tumor growth and stem cell-like properties of HCC *in vivo*. (a) Representative live images of BALB/c mice. (b, c) PPI synergistically interacted with Sora to inhibit the proliferation of xenograft tumors in BALB/c mice. (d) Body weights of BALB/c mice in the PPI, Sora, and combination groups. (e) Representative images of H&E-stained sections of the heart, liver, spleen, lung, and kidney of nude mice in the control and PPI groups. (f) Representative images of H&E staining and IHC staining for *β*-catenin, NANOG, and OCT-4 in xenograft tumors. ^∗^*P* < 0.05 and ^∗∗^*P* < 0.01*vs.* the control group; ^#^*P* < 0.05 and ^##^*P* < 0.01*vs.* the Sora group.

We further validated the antiproliferative and anti-CSC potential of PPI in nude mice. Huh-7 CSCs containing a LUC reporter were subcutaneously implanted into the right flank of nude mice to establish primary tumor xenografts. The mice bearing HCC xenografted tumors were randomly assigned into control, Sora, PPI, and PPI combined with Sora groups and treated with the indicated drugs *via* intraperitoneal injection for 4 weeks. Body weights and tumor volumes were monitored every 3 days. Live imaging of the mice was captured after an intraperitoneal injection of 150 mg/kg D-luciferin. The intravita l imaging results (Figures 8(a)–8(c)) indicated that PPI and Sora both suppressed the tumor growth in mice, and the combined PPI and Sora group showed a more obvious inhibition of tumor proliferation. There was no drop in the body weight and no death of the mice in each experimental group compared with those in the control group, revealing that PPI had no obvious adverse systemic effects (Figure 8(d)). When the mice were sacrificed after *in vivo* imaging, tumors and other organs were collected for H&E staining and IHC assays. H&E staining was used to detect the toxicity of PPI to mice. The results (Figure 8(e)) showed that there were no significant morphological changes in the heart, liver, spleen, lung, or kidney, which confirmed that PPI had no obvious toxic side effects on nude mice. H&E analysis of the tumor tissue (Figure 8(f)) further revealed that the number of cancer cells after PPI treatment was less than that in the control group, and the combination treatment showed a more significant inhibitory effect. Finally, the IHC experiment showed that the expressions of *β*-catenin, NANOG, and OCT-4 were all attenuated in the drug-treated groups compared with those in the control group, and a more pronounced effect was observed in the PPI and Sora combination group (Figure 8(f)). In summary, these results indicated that PPI significantly inhibited the tumor growth, LCSC stemness, and *β*-catenin expression.

## 4. Discussion

According to a landmark analysis, Sora, a multikinase inhibitor, improved the median overall survival of HCC patients from 8 to 11 months [39–41]. Sora was approved by the FDA as a first-line systemic drug to treat patients with advanced-stage HCC [42–44]. However, Sora frequently induces adverse events, such as diarrhea, hand and foot skin reactions, and bleeding, and leads to a poor prognosis and treatment resistance, which limits its application in the clinic. Only 30% of patients benefit from Sora because other advanced HCC patients have developed resistance to it after taking the drug, which generally occurs within 6 months [45, 46]. The mechanisms of Sora resistance are complex and undefined but are known to include increased expression of the epidermal growth factor receptor, c-Jun and AKT activation in HCC cells, epithelial–mesenchymal transition, increased numbers of CSCs, and an increase in hypoxia. Thus, new strategies are needed to eliminate HCC. Our study suggested that the stem cell-like features of HCC were inhibited by the combined action of PPI and Sora, which enhanced the tumor-killing effect. These results provide a novel insight and a basis for new therapeutic strategies for HCC.

PPI, a natural bioactive compound (steroidal saponin) that originates from the rhizome of *P. polyphylla* has previously been confirmed to significantly inhibit several cancers *via* multiple mechanisms [47]. Available studies have focused on its proapoptotic and resistance inhibition effects in HCC, non-small-cell lung cancer, and colorectal cancer cells [48]. Nevertheless, no reports have demonstrated yet that the application of PPI could affect the self-renewal or stem cell properties of HCC, thereby inhibiting the tumor growth and proliferation. More importantly, the precise mechanisms involved in the antitumor activity of PPI are not well understood. In this study, we investigated whether PPI suppresses the CSC-like properties of HCC and attempted to reveal the underlying mechanism.

Before performing the *in vivo* experiment, we first conducted a CCK-8 assay to assess the inhibitory effect of PPI against HCC cells, with the results indicating that PPI suppressed the proliferation of HepG2 and Huh-7 cells in dose- and time-dependent manners. Subsequently, a colony formation assay showed that PPI treatment exerted long-term inhibitory effects against these HCC cell lines. An assay investigating the tumor sphere-forming ability of LCSCs demonstrated that PPI treatment, with or without Sora, restrained the growth of suspended stem-like spheres that were derived from HepG2 and Huh-7 cells. Furthermore, flow cytometry indicated that the proportion of EpCAM^+^CD13^+^ cells decreased after treatment with PPI or Sora. Collectively, PPI not only restricted the proliferative capacity of HCC cells but also suppressed the characteristics of LCSCs.

It has been demonstrated in numerous studies that HCC could exhibit the aberrantly activated Wnt/*β*-catenin pathway and that *β*-catenin is involved in the regulation of canonical markers of LCSCs [49, 50]. Our study also found that the expression of *β*-catenin and of the CSC markers NANOG and OCT-4 were upregulated in LCSCs compared with that in HCC. Furthermore, both the total and fractional expression levels of *β*-catenin were downregulated in HepG2 and Huh-7 CSCs treated with PPI or Sora. In addition, an immunofluorescence assay showed that PPI and Sora suppressed nuclear accumulation of *β*-catenin in LCSCs, while PPI did not facilitate the degradation process of *β*-catenin in the presence of MG132, indicating that PPI might regulate *β*-catenin levels through proteasomal degradation. The stimulation of the canonical Wnt pathway by specific ligands deactivates GSK-3*β via* specific phosphorylation at Ser9, thereby stabilizing *β*-catenin and leading to its translocation to the nucleus [51]. Moreover, p-AKT was reported to be the upstream molecule that leads to the phosphorylation of GSK-3*β* at the Ser9 residue, resulting in the inactivation of GSK-3*β* and stabilization of the *β*-catenin protein [52]. Both PPI and Sora were found to diminish the phosphorylation of GSK-3*β* (Ser9), suggesting that PPI is a negative factor for *β*-catenin stabilization, which might lead to proteasomal degradation of *β*-catenin. In addition, p-AKT was found to be significantly inhibited by PPI or Sora in LCSCs, suggesting that the inhibition of AKT activation is involved in the PPI-induced *β*-catenin degradation. For in-depth analysis of the mechanism of action of PPI on the AKT/GSK-3*β* pathway, the AKT inhibitor LY and the GSK-3*β* inhibitor LiCl were used to suppress the activity of AKT and GSK-3*β*, respectively. LY treatment decreased the expression of *β*-catenin, and PPI exacerbated this effect, whereas PPI administration counteracted the *β*-catenin-enhanced effect of LiCl. Overall, these results showed that PPI suppressed the LCSC stemness and likely promoted *β*-catenin degradation through the AKT/GSK-3*β* signaling pathway.

To confirm that *β*-catenin is an essential mediator of LCSC stemness, LCSCs with *β*-catenin silencing were constructed using lentivirus vector transfection. Our results demonstrated that *β*-catenin silencing could effectively inhibit the proliferation of LCSCs. Furthermore, the *β*-catenin siRNA not only led to a reduction in *β*-catenin expression but also abolished the stem cell-like properties of LCSCs. Meanwhile, no significant reduction was observed in the PPI combined with siRNA group compared with that in the PPI-alone or the siRNA-alone group, indicating that PPI might abolish the stem cell-like properties of HCC by modulating the stability of *β*-catenin. Co-IP combined with a ubiquitination experiment was used to gain a deeper insight into the underlying regulatory mechanism of *β*-catenin. The results demonstrated that PPI might activate the ubiquitination process of *β*-catenin in LCSCs.

In addition, we confirmed the results of *in vitro* experiments in *in vivo* experiments. As live imaging showed, PPI, with or without Sora, could suppress the tumor growth, and the combination of PPI and Sora exerted the strongest inhibitory effect. H&E and IHC experiments also revealed that both PPI and Sora could limit the expression of *β*-catenin, NANOG, and OCT-4. To summarize, these results indicated that PPI had an anticancer effect and reduced the stemness of LCSCs via proteasomal degradation of *β*-catenin through the AKT/GSK-3*β* pathway.

## 5. Conclusions

The results of this study showed that a natural compound, PPI, activated the proteasomal degradation of *β*-catenin, thereby restraining the growth of HCC cells and blocking the activation of LCSCs, both *in vivo* and *in vitro*. To the best of our knowledge, this is the first report suggesting that PPI limits tumor sphere formation by restraining stem cell-like characteristics. This study elucidated the anticancer effects of PPI in the treatment of HCC, highlighted the importance of *β*-catenin in maintaining the stemness of LCSCs, and provided evidence for the clinical application of PPI. There are also limitations such as that flow cytometry would be a more appropriate means for measuring LCSC markers in mice. Additional studies are needed to further explore the effects of PPI on LCSCs and HCC.

## Figures and Tables

**Table 1:** Binding energies between PPI and *β*-catenin, p53, PI3K, PPAR*α*, AKT, and GSK-3*β* based on molecular docking information.

Protein	Gene	UniProt ID	PDB code	Binding energy (kcal/mol)
*β*-Catenin	CTNNBIP1	Q9NSA3	1JDH	-5.40
p53	TP53	P04637	6GGA	-5.07
PI3K	PIK3R1	P27986	1H9O	-4.66
PPAR*α*	PPARA	Q07869	3SP6	+3.98
AKT	AKT1	P31749	2UVM	-5.51
GSK-3*β*	GSK3B	P49841	1GNG	-5.32

## Data Availability

The data used to support the findings of this study are available from the corresponding author upon request.

## Abbreviations

PPI: Polyphyllin I
HCC: Hepatocellular carcinoma
LCSCs: Liver cancer stem cells
CSCs: Cancer stem cells
Sora: Sorafenib
CHX: Cycloheximide
LUC: Luciferase
DMEM: Dulbecco's modified Eagle's medium
FBS: Fetal bovine serum
P/S: Penicillin and streptomycin
BSA: Bovine serum albumin
CCK-8: Cell Counting Kit-8
PBS: Phosphate-buffered saline
qPCR: Quantitative real-time polymerase chain reaction
IHC: Immunohistochemistry
H&E: Hematoxylin and eosin.
